# The Employability and Career Development of Finance and Trade College Graduates

**DOI:** 10.3389/fpsyg.2021.719336

**Published:** 2022-01-10

**Authors:** Xiang Huang, Jiajia Cao, Guojing Zhao, Zehai Long, Guanshuang Han, Xiaowei Cai

**Affiliations:** ^1^School of International Education, Zhejiang Institute of Mechanical and Electrical Engineering, Hangzhou, China; ^2^Graduate School of Education, Peking University, Beijing, China; ^3^Teaching Quality Control Department, Wenzhou Polytechnic, Wenzhou, China; ^4^Institute of China Innovation and Entrepreneurship Education, Wenzhou Medical University, Wenzhou, China; ^5^College of Education, Zhejiang University, Zhejiang, China; ^6^School of Marxism, Zhejiang University, Zhejiang, China

**Keywords:** employability, career development, finance and trade, college graduates, multiple linear regression model

## Abstract

Employability is a vital aspect for human development in career fields. In order to explore the factors affecting the employability of finance and trade graduates in higher vocational colleges, the researchers focused on human development in educational settings and conducted a piece of quantitative research within nine higher vocational colleges. The study uses descriptive statistical analysis to demonstrate the sample structure, using *t*-test, rank sum test, and chi-square test to assess the variables. It also adopts exploratory factor analysis to identify the classification of both educational practice and employability. Then, a multivariable linear regression model was adopted to examine the relationships between three main factors as well as the employability and career development of finance and trade graduates. The findings imply that the soft skills and basic skills of finance and trade college graduates have immensely improved through educational practice; graduates with high motivation for learning could enhance their soft skills and more internships or club engagement brings stronger professional skills. Based on these results, higher vocational colleges, enterprises, policymakers, teachers, and finance and trade graduates will benefit from the findings related to the reform of educational practice for strengthening graduate employability and human development. The originality of this paper is the conceptual evolution of finance and trade college graduate employability, as well as the empirical analysis on educational practice, student engagement, and family background affecting their human development.

## Introduction

Economic development has entered a new stage with the upgrading of its quality and efficiency; in particular, the advancement of emerging industries has changed the structure of employment, putting forward new development requirements for human resources. China's higher vocational education has been in a quality improvement phase since 2003. According to China's Ministry of Education, as of 2019, there were 1,423 higher vocational colleges, 510,000 full-time teachers (Ministry of Education, 2020a), and 7.58 million undergraduate and college students, of which 3.63 million graduated from higher vocational colleges (Ministry of Education, 2020b). The growth and success of graduates in universities and colleges mainly depend on the teaching practices and the participation of graduates, rather than their family backgrounds or other factors. *The Annual Report on the Quality of Higher Vocational Education in China 2019*, jointly compiled by the Shanghai Institute of Educational Sciences and the MyCOS Institute, has made a deeper improvement in the “five-dimensional quality view,” which contains student development, school strength, development environment, international influence, and social contribution (Higher Technical Vocational Education in China, 2019). The report shows that the employment rate of higher vocational graduates continues to stabilize at 92% upon 6 months after graduation, and the monthly income increase reaches 76.2% 3 years after graduation. Higher vocational education plays an increasingly important role in expanding employment and promoting graduates' development, but it also faces the challenges of tight resources, insufficient reform policies, and urgent requirements of strong social service ability.

Rapid changes in the business environment have produced the need for the skill improvement of business graduates who ought to meet the expectations of the market (Wilton, 2011). This concern mainly focuses on the role of business education in developing employability. Finance and trade problems are multidimensional and sophisticated, requiring competitive skills to survive in business contexts. Thus, the employability of college graduates has become the core concept of educational reform and an important performance indicator for measuring the teaching quality and value-added effect of higher education (Smith et al., 2001). With the development of higher education and the diversity of career patterns in the labor market, employability emphasizes the competitiveness of college graduates, and tries to measure the contribution of higher education institutions to the employment of graduates. MyCOS's *Blue Book of Employment 2019* (Wang and Ma, 2019) analyzes the employment status of Chinese three-year vocational college graduates. In general, the report presented warnings for several majors with high unemployment rates, low salaries, and poor employment satisfaction such as legal affairs, financial management, accounting computerization, and business management. The follow-up survey report also pointed out that the absorption level of the modern service industry to higher vocational college graduates continues to rise, reaching 61.3% for the class of 2018. Meanwhile, self-employed graduates half a year after graduation were getting involved in the retail business (12.3%). Notably, majors such as business administration, finance, economy, trade, and accounting have monthly incomes of RMB 4,198, RMB 4,139, RMB 4,021, and RMB 3,685, respectively (average: RMB 4,112). From a graduate perspective, job satisfaction for the class of 2018 concerning finance and trade graduates was 65%; the main reasons for dissatisfaction of employment situation lied in “low income” (67%) and “insufficient opportunities for development” (53%). In particular, the relevancy rate between jobs and majors was only 54%, the career expectation fit rate was 44%, and the quit rate within half a year after graduation was 50%, which was higher than the average (42%), and the evaluation of the significance of the core courses was 91% with a job satisfaction of 80%. As for the finance and trade graduates of the class of 2015, the transition rate among different jobs was 60%. Correspondingly, 63% of the graduates had received a promotion 3 years after their graduation; the average time of promotion was 1.1 years and the monthly income was RMB 5,895 (average: RMB 6,005) with a 76% increase. The innovative entrepreneurship courses mainly focused on *New Retail* or other emerging areas. From a holistic perspective, finance and trade mainly focus on *finance, accounting, management, and economics (FAME)*. The average recognition rate of the class of 2018 for FAME was 2.89 (total score: 4); the degree of relevance between job and major was lower than that of non-FAME and continued its downward trend. The satisfaction of “persuading skill” development was the lowest, but it was highly important, especially for majors such as *marketing* and *finance*, knowledge and skills enhancement were relatively disappointing from the graduate's point of view. Accordingly, a suggestion from graduates was mainly emphasizing on “enhancing the internship and practical training” (60%) to encourage deeper student engagement and better teaching support to inspire their creativity.

Hence, the accuracy of the matching degree between practical training and industrial development requirements and the interaction between teachers and students both need to be highly focused and strengthened. Based on the data of 1,230 questionnaires, multiple linear regression was used to conduct empirical research to answer the following questions:

- Does the educational practice of higher vocational colleges have an impact on the employability and career development of finance and trade graduates?
- Does student engagement have an impact on the employability and career development of finance and trade graduates?
- Does family background have an impact on the employability and career development of finance and trade graduates?

The structure of this study is organized as follows. The next section discusses the factors that influence the employability and career development of finance and trade graduates as the basis for our hypotheses. Section Methods explains the methods, including the research design, questionnaire and sampling techniques, participant and procedures, variables and measures, multiple linear regression model used in this study, and the reliability and validity analysis. Section 4 describes the empirical research process and analysis results. It concludes with a discussion, conclusions, implications for theory, implications for practice and limitations, and further research opportunities.

## Literature Review and Research Hypotheses

### Graduate Employability

Employability-related research has been increasing in recent years. “Career” refers to the experience and aggregation of all activities during work, which is related to the progress and development of individuals in the professional field (Greenhaus et al., 2009). It also refers to the promotion of individuals in an established organization and professional institution based on their performance. The term *boundaryless career* breaks the organization's guarantee of long-term or lifetime employment, emphasizing the employability enhancement to ensure continuous employment across different organizations, which indicates the uncertainty and variability of personal career development (Arthur, 1994; Sullivan, 1999; Hall, 2004). “Boundaryless” is driven by personal will in the process of accumulating professional capital, such as value, skills, experience, and employability.

The concept of employability first appeared in Britain in the early twentieth century. It can also be called employment ability, core ability, key competence, or employment skills. With the development of higher education and the increasing diversification of career forms in the labor market, the concept connotation of employability has gradually developed and extended. The Confederation of British Industry (CBI) defines employability as a combination of attributes, skills, and knowledge that all graduates or ready-to-work participants should have to demonstrate their capability in the labor market and bring benefits to themselves, organizations, and society (CBI/Nation Union of Students, 2011). The Organization for Economic Co-operation and Development (OECD) points out that besides knowledge and skills, employability also includes teamwork ability, ability to handle non-routine procedures, ability to communicate and solve problems, and even transferability (Pont, 2001). *The Employability Skills Framework* from the Australian Government defines employability as the ability of individuals to achieve employment, career development, and potential realization, which includes communication ability, problem-solving ability, initiative and entrepreneurial spirit, planning and organization ability, self-management ability, and scientific and technological skills (Mclean et al., 2012). Employability refers to a series of graduates' achievements, such as skills, understanding, and personality characteristics that are beneficial to themselves, the labor market, community, and economic development. It is related to the ability to successfully assume a career role and transfer among occupations, so as to develop their own career sustainability (Hillage and Pollard, 1998; Yorke, 2006). Business organizations would recruit graduates with practical work skills and experience. Cumming (2010) supported this idea by presenting the demands of employers and the labor market, which boost the enhancement of graduates' academic skills as well as practical skills by adding employability skills to college curricula. There is a lack of empirical evidence, especially for finance and trade graduate employability. Presently, the goal of employment policy is changing from “employment rate” to “employability,” which means the shift from quantity to quality has posed an urgent question, which is how to effectively shape and enhance the employability of graduates through training and practicing.

Research on the influencing factors of employability presents a variety of different measurement dimensions. McQuaid and Lindsay (2005) divided the influencing factors of employability development into three aspects: personal factors, individual environment, and external factors. The personal factors include employment ability, skills and characteristics, demographic characteristics, health, job search, adaptability, and flexibility; individual environment includes family environment, work culture, and resource acquisition. External factors mainly focus on demand factors and supporting factors to obtain employment. Among them, skills and qualities for employment ability mainly refer to basic transferable skills (document processing, writing, calculation, and oral expression skills), key transferable skills (reasoning, problem solving, adaptability, work process management, personal task and time management, flexibility, basic information and communication skills, interpersonal communication, and emotional and aesthetic customer service ability) and high-level transferable skills (teamwork, business thinking and logic, continuous learning, vision, specific work ability, and enterprise-related skills; McQuaid and Lindsay, 2005). However, in the process of graduates' participation in employment, the educational attribute will play a more obvious role than the social attribute in their choice of employment mode and employment results, especially in the accumulation of graduates' human capital. Clarke (2018) comprehensive model of graduate employability focuses on the influence of perceived employability by graduates' human capital, social capital, and individual behavior and characteristics, and then affects students' engagement in career search, job maintenance, and job transformation. At the same time, labor market factors will also play a role in perceived employability and employment success. Specifically, as an important part of higher education, skill training shapes graduates' human capital, such as problem-solving ability, critical thinking, teamwork ability, and so on (Clarke, 2018). What's more, empirical research on factors affecting business graduate employability show that both soft skills and technical skills are positively related to employability, and social mobility factors also play a significant role in employability (Hossain et al., 2020).

Therefore, researchers have carried out different element constructions of employability from various angles, showing different contents and dimensions. The research on employability has experienced a process of developing from simple research to complex and integrated research. It then attaches importance to the competitiveness of college graduates, pays attention to quality, and tries to measure the contribution of higher education institutions to graduate employability. However, while most of the current research assumption is based on the employability of stable organizational structure and career acquisition within one organization, more analysis and measurement of the connotation of employability should be carried out around the idea of boundaryless careers. By combining McQuaid's analysis on the structure of employability as well as the Peking University's national survey, this study emphasizes on three pillars of the employability: soft skills, professional skills, and basic skills.

### Educational Practice and Employability

With the increasing issue of unemployment for college graduates, how to strengthen the relevance and effective connection between higher education and the labor market has become a policy issue (Teichler, 2007). And the employability of graduates has become an important performance index to measure the teaching quality of colleges and universities (Smith et al., 2001), and the top-ranking factors, i.e., strong employability, job or internship experience, and job-seeking skills, are directly related to quality employment (Yue and Zhou, 2019). Early in the 1970's, Astin's (1970) I-E-O Model emphasized on college influence on students' achievement, which indicates the *input* element as the students' personal characteristics before entering higher education institutions, including demographic characteristics, family background, educational expectations, academic qualifications, and social experience which directly and indirectly affects the *output* elements through environmental factors; *environment* elements refer to the various experiences received during their study in colleges or universities, including college characteristics, teaching staff, learning courses, learning support, and student participation. The *output* element, that is student achievement which refers to the cognitive and emotional abilities obtained through higher education, include personality, knowledge, skills, attitudes, values, beliefs, and behaviors after graduation. It could be linked to the *Good Teaching Practice*, which has a positive effect on students' academic achievement (Pascarella and Terenzini, 2005).

Harvey and Bowers-Brown (2003) research (2003) also shows that the shaping and improvement of students' employability is closely related to the activities of teaching and learning from colleges. For instance, innovative teaching methods such as “case study,” “empirical practice,” and “problem-based learning” are introduced into the curriculum design to enhance student employability (Fallows and Steven, 2000); students' professional ability and general ability could be improved through curriculum module and teaching process design (Yorke, 2006). Meanwhile, contextualized performance enacts skills in a variety of authentic settings and challenging circumstances, which establishes links with literature on curriculum and pedagogy in order to enrich the capability of graduates (Cumming, 2010). Therefore, employment-oriented curriculum design and learning activities could solve the current worries about graduate employment issues (Ahmed et al., 2015; Baek and Cho, 2018). Learning transferability and articulating skills of students could be fostered, operationalized, and optimized when delivered as part of work-based/work-related programs of study (Ehiyazaryan and Barraclough, 2009). And universities are now adopting internships, work placements, and international study in their degree programs with the aim of enhancing graduate employment prospects (Clarke, 2018). Meanwhile, a research model to reveal that graduate employability can be improved through *university and industry collaborations* (UIC) that provide students with mentoring opportunities and exposure to relevant training was constructed (Aliu and Aigbavboa, 2020). Graduates who have accepted the problem-based teaching pattern could enhance their critical thinking and employment-related competencies (Baek and Cho, 2018; Liu et al., 2020); the design of learning context is highly correlated with employability and stimulates positive abilities. Similar research results like *pedagogy for employability* (POE) has a positive impact on students' *absorptive capacity* (AC; Li et al., 2020) and also proves the importance of educational practice, which enhances graduate employability of identifying, digesting, and applying the process into their available knowledge. Employability skills could also be developed through understanding the conditions and standards of the curriculum and teaching activities of teachers, which leads to the successful creation of flipped learning (Peng et al., 2021).

Thus, researchers have focused on educational practice and carried out in-depth analysis at the college perspective. Majors and curriculum, mode of talent cultivation, teaching methods and processes, teachers and tutors, employment guidance, and extracurricular activities have a positive impact on shaping graduate employability. And cooperation between colleges and enterprises, like training and internships, could greatly enhance graduates' practical skills as well as the awareness of career adaptation and development. However, there is a lack of a detailed and clear construction of the pathway, which could not reveal the curriculum, teaching practice, graduates' diverse experience, and education-related practice with different characteristics that enhance graduate employability, thus inhibiting the promotion of constructive recommendations for effective production-education integrative reform. Based on the above analysis, we propose hypothesis 1.

*Hypothesis 1. Educational practice has a significant positive impact on the development of finance and trade graduate employability*.

### Student Engagement and Employability

Designing academic programs around the various stages of the learning process could not only be effective in delivering employment-related knowledge and skills, but also attract, engage, and retain enthusiastic and talented students to the field (Senior et al., 2014). Xie and Derakhshan (2021) introduced and defined seven instances of positive teacher interpersonal communication behaviors (teacher care, immediacy, clarity, credibility, rapport with students, praise, and confirmation), which positively predict students' academic outcomes like motivation, engagement, involvement, success, and learning. There are several important theories of student engagement in study or social activities at colleges that have emerged. *Theory of Involvement* explains the dynamic development process of students during their study in colleges and universities. Compared with students' extracurricular participation, it is found that their academic participation has more impact on students' achievement (Astin, 1984, 1993). *Student Integration Theory* is divided into social integration and academic integration. Social integration is students' cognition of peer interaction and teacher-student interaction, and academic integration is formal or informal empirical cognition formed by students' interaction with teachers or peers inside and outside the classroom (Tinto, 1993). *Student Engagement Theory* includes the time and energy invested by students in academic and institutional activities, and considers how colleges and universities attract students to participate in activities to achieve academic experience and satisfactory outcome (Kuh et al., 2007).

Student engagement in Higher Education is the focus of considerable research (Qureshi et al., 2015). The concept of student engagement has been viewed as multi-dimensional with cognitive, behavioral, and emotional aspects with historic roots in study on student involvement (Trowler, 2010). The data from the OLT project *Developing Graduate Employability* (Jollands et al., 2014) has identified the specific employability skills for graduates with a broader set of competencies, such as communication, teamwork, and leadership skills, that go beyond subject-specific knowledge (Arsenis et al., 2021). It also involves a broad range of employability skills and knowledge learned in many contexts and through a range of experiences (Araujo et al., 2015).

The Belonging Project (Araujo et al., 2015) at RMIT University in Melbourne explored and evaluated five pilot initiatives to improve and support student engagement in disciplinary communities, including private study, group work, socializing, and engaging in a range of cohort events, including student-led exhibitions and industry events, which proved that student engagement would help to develop professional skills in the creation and promotion of their work. The pro bono project (Blandy, 2019) at the University of Sheffield also verified the benefits of students participating in pro bono activities for professional skills and employability. Similarly, a new “pitch” assessment (Smith, 2012) helped students to present their pitch through a combination of video, oral, and poster presentations and supplementary documents, which successfully encouraged them to identify their relevant skills and experiences of future career options. And video group assessment (Arsenis et al., 2021) has been viewed as a positive and engaging but also challenging experience for students to collaborate and develop teamwork and communication skills for future employment. Therefore, based on the theories of student engagement, researchers have emphasized on graduates' participation in educational practice and evaluated various ways to improve and support their engagement both in learning and practicing. However, there are many dimensions of student engagement, and they have different effects on the development of graduate employability and their career, hence, more detailed exploration and analysis are required to find out more feasible ways. Based on the above analysis, we propose hypothesis 2.

*Hypothesis 2. Student engagement has a significant positive impact on the development of finance and trade graduate employability*.

### Family Background and Employability

Human capital is regarded as an important factor affecting the starting salary and occupational attainments of employment. Researchers paid attention to the influencing factors of college graduate employability from the perspective of pedagogy and found that the factors of family economic conditions, parents' education level, and social and political status play an important role in employability. Based on status attainment model, Ma and Zhang (2019) put forward that family background, as an ascribed factor, means that the economic, social, and cultural capital of a family will be transformed through higher education and thus affect graduate employability. Shi and Wang (2018) found that graduate employability varied in gender, place of origin, and family economic status in China. Yue (2018) confirmed that the development of urban graduates' ability is more significant than rural graduates. Pellizzari (2010) found that family networks could influence the possibility of getting a fine job or of receiving various forms of occupation across countries. According to Lezotte and Snyder (2010), graduates' family background had a pivotal contribution to academic performance that further paved the way toward success in getting a job.

Meanwhile, empirical evidence has been provided to show that family background was significant in graduate employability (Ciriaci and Muscio, 2010). An econometric analysis (Leoni, 2011) documented that the cultural background of the family of origin tends to assume greater importance than the formal educational level acquired. The research regarding university students in China (Mok and Wu, 2015) perceived that family background, socio-economic status, social networks, and social capital would have significant influence on graduate employment, although it showed that family background was not significant in explaining Italian graduates' labor market performances (D'Hombres et al., 2008). Critical survey data of undergraduates from 20 universities in China (Ma and Zhang, 2019) revealed that graduates with more advantaged family backgrounds have stronger employability, because graduates of advantaged families with a higher starting point, richer educational resources, more extensive social relations, and better family upbringing. Thus, they have a greater chance to enter well-known colleges and universities, which reflected the cultural reproduction mode of educational inequality. Therefore, current research indicates that family background, i.e., family background, family networks, and socio-economic status, is also an important aspect that positively influences graduates' employment. However, as the top-ranking factor of employment, the term of employability has been defined as “a set of achievements, understandings, and personal attributes that make individuals more likely to gain employment and to be successful in their chosen occupations” (Yorke, 2006), thus, more in-depth and targeted analysis is needed to understand the impact of family background on the specific abilities of graduate employability. Based on the above analysis, we propose hypothesis 3.

*Hypothesis 3. Family background has a significant positive impact on the development of finance and trade graduate employability*.

**Figure 1:**
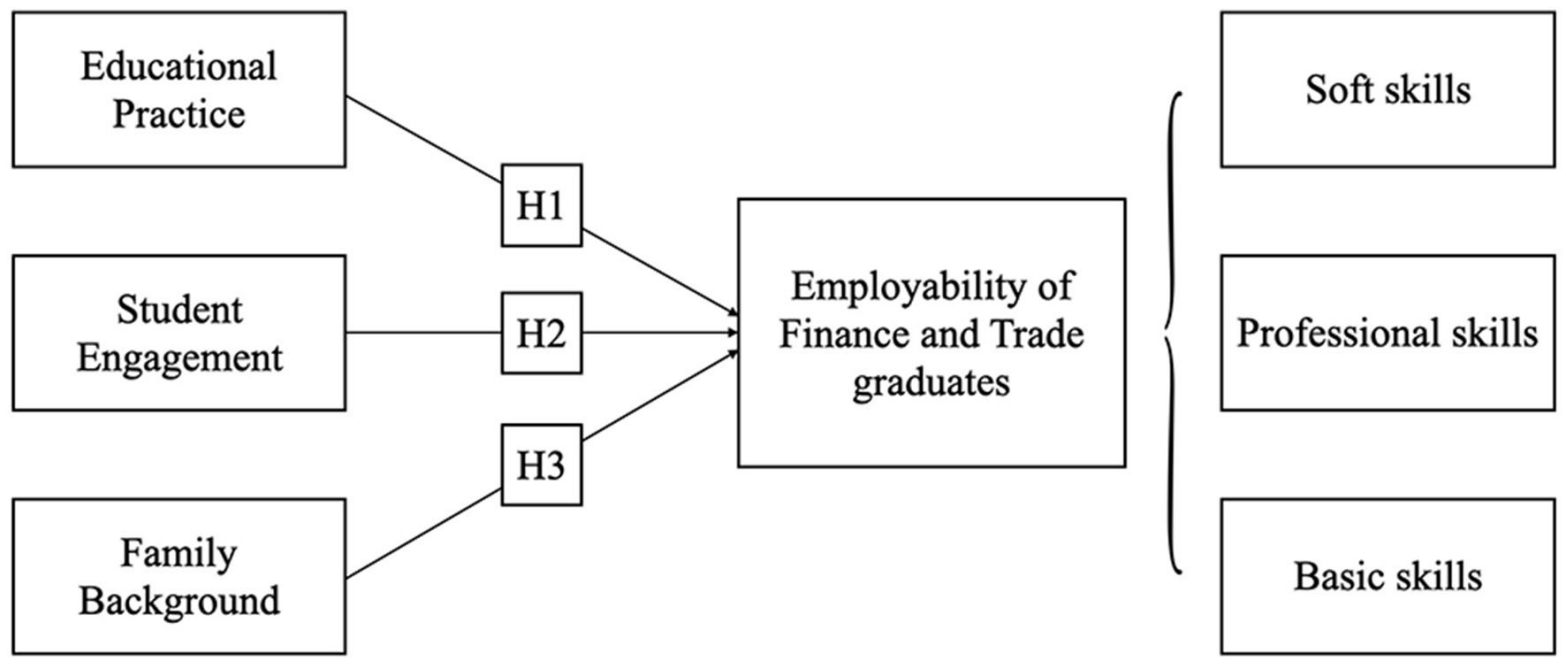
Research model.

This research model focuses on the impact of educational practice, student engagement, and family background on finance and trade graduate employability, which consists of three pillars: soft skills, professional skills, and basic skills (Figure 1).

## Methods

### Research Design

The research mainly focused on the employability and career development of finance and trade college graduates. By using descriptive statistical analysis, this study demonstrated the sample structure. It adopted exploratory factor analysis to identify the three pillars of both educational practice and graduate employability; then, a multivariable linear regression model was adopted to discuss the impact of educational practice, student engagement, and family background on employability development of finance and trade graduates. Finally, we reported the preliminary findings, particularly what we have learned about the significance of enhancing the quality of vocational college education.

### Questionnaire and Sampling Techniques

Based on the existing relevant research and literatures, this paper adopted *National Employment Survey of College Graduates* questionnaire surveys in 2019 conducted by *Economics of Education* at Peking University (Yue and Zhou, 2019), and focuses on the finance and trade graduates of higher vocational colleges. Starting from 2003, this nation-wide survey has been conducted every other year in June or July. The survey subjects comprise the graduating class of the given year, and in each survey, 500–1,000 questionnaires were distributed to each sampled institution of higher education at a certain ratio, based on the disciplinary fields and level of educational attainment of the graduates. The collected data for all surveyed institutions of higher education is representative as a national sample of college graduates. The questionnaires contain four sections addressing the basic information of college graduates, as well as their job-seeking status, employment status, and status of receiving higher education. The overall reliability and validity of the survey were good, and the Cronbach's α coefficient of the scale used conforms to the standard (>0.80). Meanwhile, in terms of content validity, the Peking University questionnaire used in this paper has been improved over more than 10 years of survey practice and repeated discussion by experts, and can be considered to have good content validity. This questionnaire survey in 2019 included 32 colleges and universities in 17 provinces, regions, and cities in the east, central, and western regions of China. A total of 16,571 valid questionnaires were collected. Among the valid samples, according to the educational level, vocational college graduates accounted for 23.4%, undergraduates accounted for 62.6%, master graduates took up 13.0%, and the remaining 1.0% was occupied by doctoral graduates.

### Participants and Procedures

In this study, the survey was conducted with 1,230 (65.3% female, 40.1% came from one-child families) graduates within nine higher vocational colleges majoring in finance and trade, with a mean age of 21 years.

In order to conduct the research in an ethically accountable way and ensure that the research leads to beneficial outcomes, prior to participation, graduates were informed about the goals of the study, duration, procedure, and confidentiality of their data. Participation in the study was voluntary, informed consent was assured, and graduates did not receive compensation for their participation. Participants were assured that all of their responses would remain confidential. All graduates were asked to evaluate their ability-related evaluations toward educational practice, as well as their participation in both academic and social activities, and others.

### Research Variable and Measures

In this study, we measured both independent and dependent variables from the survey. The dependent variable was the value-added abilities of finance and trade graduates in higher vocational colleges; it was measured using a 5-level Likert-type scale with the anchors of 1 = “not much” through 5 = “a great deal.” A 34-items self-report question was used: “How will you assess the following abilities development (value-added) during your college life?” Table 1 shows the graduates' responses to the “Graduate ability development” scale in this study. By comparing the answers for their employability development, the mean value was basically above 3.89 on the whole and only two items were lower than 3.89; meanwhile, there were some differences in the standard deviation, reflecting that the graduates' answers had a certain degree of differentiation, and the responses were good.

**Table 1:** Descriptive statistical analysis of graduates' ability development.

**No**.	**Variable**	**Mean**	**Standard deviation**	**No**.	**Variable**	**Mean**	**Standard deviation**
1	Broad general knowledge	4.06	0.802	18	Attention to detail	3.98	0.846
2	Professional knowledge	4.01	0.797	19	Time management	3.94	0.839
3	Methodological knowledge	3.97	0.829	20	Relevance of work	3.94	0.848
4	Foreign language skill	3.76	0.964	21	Manipulative skill in professional areas	3.93	0.843
5	Computing skill	3.89	0.884	22	Independent working ability	3.96	0.835
6	Financial literacy	3.89	0.885	23	Teamwork ability	3.98	0.853
7	Knowledge of complex social, organizational, and technological system	3.93	0.871	24	Flexibility	3.95	0.860
8	Planning, coordination, and organizational skill	3.97	0.808	25	Confidence, decisiveness, firmness	3.97	0.853
9	Sorting out opinions and information processing skill	3.93	0.820	26	Concentration of attention	3.98	0.863
10	Statistical and data-processing skill	3.91	0.853	27	Loyalty and integrity	4.04	0.854
11	Problem-solving skill	3.97	0.830	28	Worldwide vision	3.84	0.918
12	Learning skill	3.99	0.820	29	Language competence	3.92	0.893
13	Self-evaluation skill	3.93	0.829	30	Written communication skill	3.94	0.887
14	Innovation skill	3.92	0.848	31	Reading comprehension skill	3.95	0.859
15	Critical thinking skill	3.89	0.871	32	Tolerance	4.00	0.863
16	Negotiation and decision-making skill	3.92	0.855	33	Leadership	3.92	0.895
17	Ability to work under pressure	3.93	0.866	34	Responsibility	4.01	0.871

Based on this, the independent variables identified five main categories, namely demographic characteristics, family background, student engagement, human capital, and college educational practice, to analyze the influencing factors of the employment development mechanism. Accordingly, this study considered student engagement in two aspects: academic engagement and social engagement. The learning length, minor courses/dual degrees, and qualification certificates are attributed to academic engagement, while social engagement emphasizes internship engagement, club activities engagement, social networking length, and student–related matters (Table 2).

**Table 2:** Research variables analysis.

**Variable**			**Definition**
Demographic characteristics	Gender		Male, Female (control group)
	Family structure		One child (control group), More than one child
Family background	Family location		Provincial capital and municipalities, Province administrative cities, County towns, Townships, Villages (control group)
	Family yearly income per capita		Above RMB 50,001, RMB 20,001-50,000, RMB 10,001-20,000, RMB 5,001-10,000, RMB 3,001-5,000, Below RMB 3,000 (control group)
	Parents' education years		Master (19 years), Undergraduate (16 years), Vocational college (15 years), High school or technical secondary school (12 years), Junior high school (9 years), Elementary (6 years), Illiterate or semi-illiterate (0 year)
	Parents' occupation		Managerial and technical staff, Non-managerial and technical staff (control group)
Student engagement	Academic engagement	Learning length	Continuous variable: Hours per day
		Minor courses/dual degrees	Yes, No (control group)
		Qualification certificates	Obtained (language, computer and vocational skills), Not obtained (control group)
	Social engagement	Internship engagement	Continuous variable: percentage in spare time
		Clubs activities engagement	Continuous variable: percentage in spare time
		Social networking length	Continuous variable: hours per day
		Student-related matters	Student leader, not involved (control group)
Human capital	Academic scores	Top 25% in major, Middle 25–50% in major, Bottom 50% in major (control group)
	Scholarships		Obtained, not obtained (control group)
College educational practice	Professional education		Factor score
	Transferable education		Factor score
	Teaching resources support		Factor score
Employability	Soft skills		Factor score
	Professional skills		Factor score
	Basic skills		Factor score

Educational practice was measured using a 5-level Likert-type scale with the anchors of 1 = “poor” through 5 = “excellent,” and a 12-items self-report question: “How do you assess the learning conditions and opportunities that the college provides?” As for student engagement, seven items included in the questionnaire were used to assess graduates' engagement in both academic and social activities. Two example questions are “How much time do you usually spend on study?” and “Have you participated in student-related matters?” And two items were used to assess graduates' human capital: academic scores and the scholarships. As for family background, four items were assessed, i.e., “Where is the location of your family?” and “What is your family yearly income per capita?” The demographic characteristics were assessed by identifying the graduates' gender and their family structure.

### Multiple Linear Regression Model

Multiple linear regression (MLR) is an extension of simple regression with the difference being the number of predictor variables employed (Allison, 1999). It is a statistical technique that uses several explanatory variables to predict the outcome of a response variable which aims to model the linear relationship between the independent variables and the dependent variables.

The research method used in this study was a quantitative research approach. It was carried out with 1,230 finance and trade graduates from nine higher vocational colleges in China. The finance and trade discipline contains nine majors: finance and tax, banking, financial accounting, statistics, economy and trade, business administration, marketing, e-commerce, and logistics. Through the reliability and validity test of the data, it was found that the overall reliability and validity of the data were good, and the current study uses descriptive statistical and factor analysis to demonstrate the sample structure as well as the relationship among variables by using the *t*-test, rank sum test, and chi-square test. Most importantly, we adopted a multivariable linear regression model to examine the proposed hypotheses. By designing multivariable linear regression models, the method emphasizes the explanation of employability development affected by several independent variables; in particular, it explores the influence of educational practice and student engagement.

Multicollinearity means that it is difficult to accurately estimate the model because of the existence of an accurate correlation or high correlation between explanatory variables in the linear regression model, that means when two or more of the independent variables are substantially correlated amongst each other, the regression coefficients and statistical significance become unstable and less trustworthy (Morrow-Howell, 1994). Tolerance, variance inflation factor (VIF), eigenvalue, and condition index are the four indicators used to judge the existence of multicollinearity. This is done by increasing the sample, screening the combined fitting model, removing the secondary collinearity factors that have more missing values and larger measurement errors, providing principal component factors to replace the original variables, and carrying out path analysis to solve the problem of multicollinearity (Smith and Sasaki, 1979). For example, VIF measures the number of inflated variances caused by multicollinearity. The VIF values in this regression analysis were <10, it is considered that the collinearity among explanatory variables is weak after the multiple collinearity test; thus, the method of metrological regression was adopted for further study. Regression results were obtained by regression with soft skills, professional skills, and basic skills as dependent variables. The significant probability of the regression model is <0.01, which indicates that there is a highly significant linear relationship between explanatory variables and explained variables.

### Reliability and Validity Analysis

As for employability, the reliability can be reflected by calculating the Cronbach's alpha value of each scale, and SPSS26.0 was applied in this research. According to the survey, the Cronbach's alpha of the whole scale of 34 variables of finance and trade graduate employability development for the question, “How will you assess the following abilities development (value-added) during your college life?” was 0.984; the Cronbach's alpha for soft skills was 0.967, the Cronbach's alpha for professional skills was 0.968, and the Cronbach's alpha for basic skills was 0.927. This means that the internal consistency of both the whole scale and the three subscales is satisfactory. Hence, the reliability of the scale and sample is high. The validation factor analysis was then used to further explore the reliability and validity. In the questionnaire, the 34 variables for the question were subjected to principal component analysis, and the Kaiser-Meyer-Olkin (KMO) test yielded 0.984, and the Bartlett's test of sphericity yielded a chi-squared value of 36462.990, with a small associated significance level (sig.) of 0.000 (Table 3). The factors with eigenvalues around and above 1 resulted in 22.481, 1.559, and 0.810, which explained 29.029, 23.669, and 20.390% of the variance, respectively. This implies that finance and trade graduate employability could be condensed into three clusters with these eigenvalues. These three clusters of employability variables had a cumulative of 73.088% of the total significance. The correspondence between the scale factors is consistent and factor loading values are all above 0.5, with no cross-factor phenomenon. Subsequently, these three clusters were renamed based on the components of each cluster, resulting in the development of graduate employability.

**Table 3:** Exploratory factor analysis for finance and trade college graduate employability.

**Kaiser-Meyer-Olkin (KMO) and Bartlett's Test of Sphericity**			
KMO Measure of Sampling Adequacy.			0.984
Bartlett's Test of Sphericity	Approx. Chi-Square		36462.990
	df		561
	Sig.		0.000
**Rotated Component Matrix**			
	**1**	**2**	**3**
32. Tolerance	0.784		
31. Reading comprehension skill	0.775		
34. Responsibility	0.768		
27. Loyalty and integrity	0.750		
29. Language competence	0.744		
30. Written communication skill	0.744		
25. Confidence, decisiveness, firmness	0.713		
26. Concentration of attention	0.710		
33. Leadership	0.696		
24. Flexibility	0.681		
28. Worldwide vision	0.628		
23. Teamwork ability	0.626		
22. Independent working ability	0.576		
13. Self-evaluation skill		0.688	
15. Critical thinking skill		0.687	
16. Negotiation and decision-making skill		0.682	
17. Ability to work under pressure		0.650	
14. Innovation skill		0.633	
11. Problem-solving skill		0.628	
12. Learning skill		0.609	
9. Sorting out opinions and information processing skill		0.608	
19. Time management		0.601	
10. Statistical and data-processing skill		0.592	
20. Relevance of work		0.575	
21. Manipulative skill in professional areas		0.573	
8. Planning, coordination and organizational skill		0.571	
18. Attention to detail		0.559	
2. Professional knowledge			0.772
4. Foreign language skill			0.748
1. Broad general knowledge			0.733
3. Methodological knowledge			0.712
5. Computing skill			0.697
6. Financial literacy			0.669
7. Knowledge of complex social, organizational, and technological system			0.623

*Extraction Method: Principal Component Analysis*.

*Rotation Method: Varimax with Kaiser Normalization*.

As for educational practice, in the questionnaire, 12 items for the question, “How do you assess the learning conditions and opportunities that the college provides?” used a 5-level Likert-type scale with the anchors of 1 = “poor” through 5 = “excellent”. We classified 12 items into three orientations: “professional education,” “transferable education,” and “teaching resources support” (Table 4). First, professional knowledge learning, professional skills cultivation, opportunities for joining in tasks or projects, and chance of major conversion are mainly focusing on the practice in the classroom around professional learning and development in higher vocational colleges, which is summarized and named “professional education;” second, general education courses (including curriculum and teaching quality), opportunity for off-campus internship, chance for participating in on-campus clubs, and interdisciplinary learning are all related to the transferable and transformative abilities' development of graduates, at the same time, these education opportuinities emphasize the cultivation of graduates' both cognitive and non-cognitive abilities, which is summarized and named as “transferable education.” Lastly, career guidance, library facilities and collections, teaching staffs' capabilities, and teaching-facilitative conditions are all useful support provided by the colleges to enhance graduates' competencies in the workplace, focusing on professional norms, rules, and literacy; therefore, it is named “teaching resource support.”

**Table 4:** Exploratory factor analysis for educational practice.

**College learning conditions, opportunities, and resources**	**Orientation**
Professional knowledge learning	Professional education
Professional skills cultivation	
Opportunity for joining in tasks or projects	
Chance of major conversion	
General education courses (including curriculum and teaching quality)	Transferable education
Opportunity for off-campus internship	
Chance for participating in on-campus clubs	
Interdisciplinary learning	
Career guidance	Teaching resources support
Library facilities and collections	
Teaching staffs' capabilities	
Teaching-facilitative conditions	

The reliability of educational practice can also be reflected by calculating the Cronbach's alpha value. Within this study, we adopted finance and trade graduates' evaluations of learning conditions, opportunities, and resources of the college to identify the level of both cultivation and educational practice. According to the survey, the Cronbach's alpha of the whole scale of 12 variables of the learning conditions and opportunities that the college provides was 0.958; the Cronbach's alpha for professional education was 0.884, the Cronbach's alpha for transferable education was 0.886, and the Cronbach's alpha for teaching resources support was 0.881. This means that the internal consistency of both the whole scale and the three subscales is satisfactory. Hence, the reliability of the scale and sample is high. As for validity analysis, four items in each orientation were subjected to principal component analysis, and the KMO test indicates a high internal consistency and reliability of the research instrument (*P* < 0.001). The KMO test of all 12 items yielded 0.962, which was adequately above the 0.5 threshold (Hair et al., 2010). Moreover, Bartlett's test of sphericity yielded a chi-squared value of 11195.625 with a small associated significance level (sig.) of 0.000, which indicates data suitability for exploratory factor analysis (Tabachnick and Fidell, 2007). According to Moser and Kalton (1971), the values are between 0 and 1, and the closer the value is to 1, the more reliable the research instrument. The factor with an eigenvalue above 1 resulted in 8.207, which explains 68.4% of the variance, and the factor score represents the overall educational practice level. The Kaiser-Meyer-Olkin (KMO) test of professional education yielded 0.838, the Kaiser-Meyer-Olkin (KMO) test for transferable education yielded 0.841 while the Kaiser-Meyer-Olkin (KMO) test for teaching resources support yielded 0.829, which are all higher than 0.7, indicating a strong bias correlation of the variables, while the calculated chi-square values of Bartlett's statistic for each scale are all significant; the correspondence between the question items of each scale and the scale factors is consistent with the study's expectation.

## Analysis of the Research Process and Results

### Descriptive Statistical Analysis

After descriptive statistical analysis of variables, the data showed 14.8% of graduates were from “Provincial capital and municipalities” families, 16.1% were from “Province administrative cities” families, 30.8% were from “County towns,” 10.4% were from “Townships,” while 27.9% were from “Villages.” As for family yearly income per capita, 13.3% graduates were from families with yearly income per capita “above RMB 50,001,” 10.0% were between “RMB 20,001–50,000,” 16.9% were between “RMB 10,001–20,000,” 21.7% were between “RMB 5,001–10,000,” 19.4% were between “RMB 3,001–1,000” while 18.7% graduates' family yearly income per capita was “Below RMB 3,000.” As for parents' education years, 15.5 and 18.8% graduates' fathers and mothers had received more than 15 years' education, 24.7 and 27.6% graduates' fathers and mothers graduated from “High school or technical secondary school,” 29.5 and 32.0% just finished “Junior high school,” while 30.3 and 21.6% graduates' fathers and mothers had received <6 years' education. As for parents' occupation, 23.6% of graduates' fathers were “Managerial and technical staff” while the percent for mothers was 16.6%.

The variable of student engagement contains graduates' academic engagement and social engagement. As for academic engagement, the average “Learning length” of finance and trade graduates was 5.7 h per day and 52.3% graduates invested more time than the average; 13.3% graduates had participated in “Minor courses/dual degrees” learning and 85.6% graduates had obtained “Qualification certificates (language, computer, and vocational skills).” As for social engagement, the average percentage of “Internship engagement” in their spare time was 41.32 and 46.4% graduates had spent more time on internships than the average; the average percentage of “Clubs activities engagement” in their spare time was 13.68 and 60.5% graduates had spent less time on clubs activities than the average. The average of “Social networking length” was 3.75 h per day and 39.7% graduates spent more time than the average; finally, 46.7% graduates were “Student leaders” while 53.3% were not involved in “Student-related matters.” As for the variable of human capital, 35.2% graduates' academic scores were in the “Top 25% in major,” and 45.3% were “Middle 25–50% in major,” while 19.5% were “Bottom 50% in major.” Meanwhile, 31.3% graduates had obtained scholarships while 68.7% had not.

According to the finance and trade graduates' evaluation of learning conditions and opportunities offered by colleges, there are five levels to the assessment: “poor,” “fair,” “average,” “good,” and “excellent.” For professional education, there was more than 70% of the high evaluations (good and excellent) from the graduates for professional knowledge learning, professional skills cultivation, and opportunity for joining in tasks or projects, with an overall figure of 67.4% of average and good for the chance of changing majors. In the aspect of transferable education, the overall evaluation of “general education courses” is high, with the total proportion of good and excellent being 75.7%, while the evaluation of “interdisciplinary learning” is only 63.5%. As for teaching resources support, 30.4% of the graduates think that “career guidance” is not good enough (poor, fair, and average) and needs to be improved.

**Figure 2:**
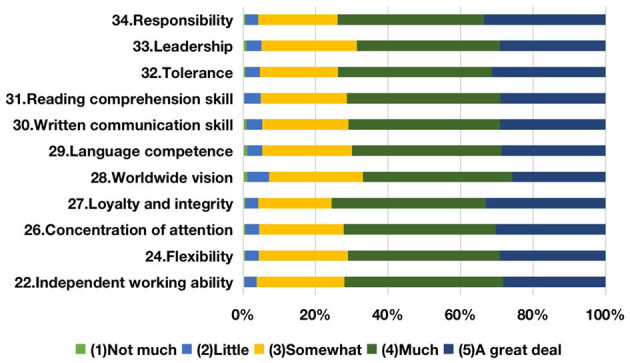
Development of graduates' soft skills.

**Figure 3:**
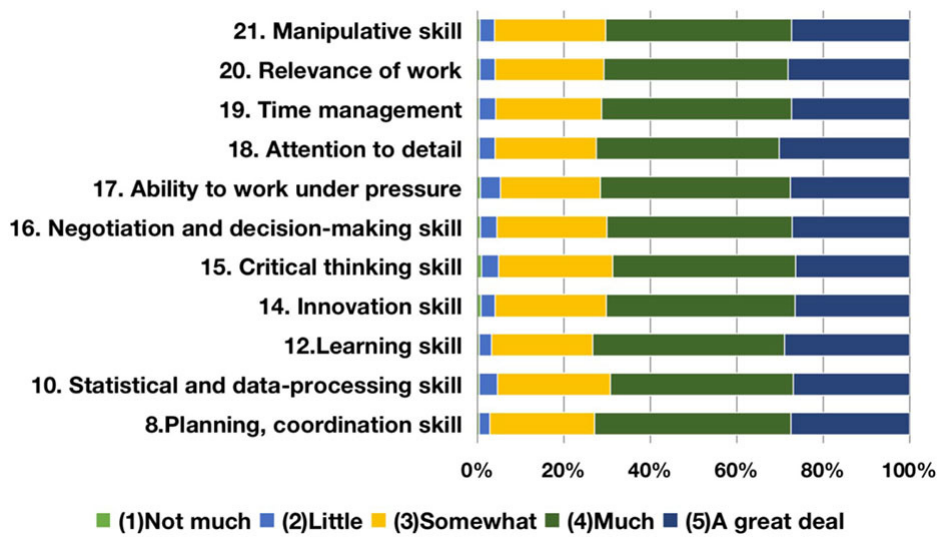
Development of graduates' professional skills.

**Figure 4:**
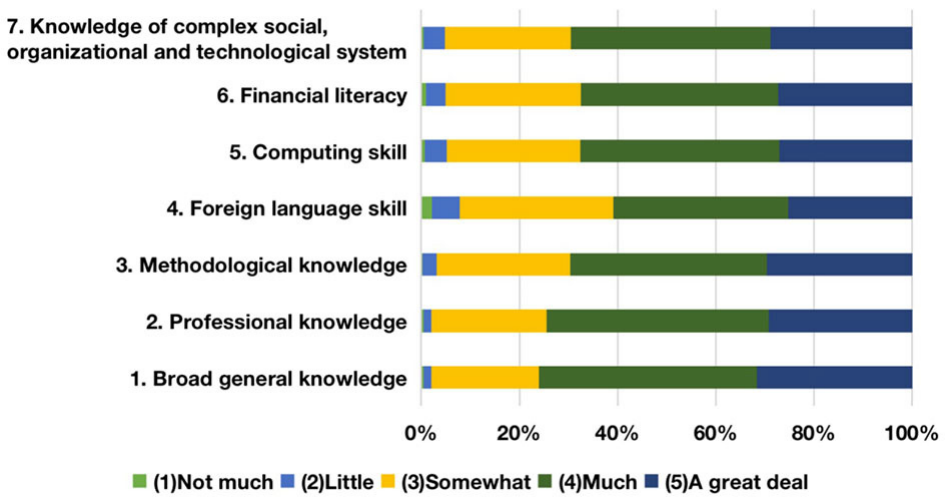
Development of graduates' basic skills.

As for employability, based on these components, this study renamed the first cluster “soft skills” (Figure 2). It contains personal inherent spirit and moral behavior, as well as the capability to communicate and offer empathy. These two kinds of measurement variables not only demonstrate the qualities and capabilities needed to fulfill tasks, but also a sense of teamwork that survives in a competitive business environment. According to the finance and trade graduate evaluation of employability development during college time, the top three items are “loyalty and integrity,” “responsibility,” and “tolerance” with the total proportions of “much” and “a great deal” at 75.5, 73.9, and 73.7%, respectively; notably, “worldwide vision” and “leadership” are relatively less developed according to the survey. The second cluster, “professional skills” (Figure 3), contains the capabilities required during the career period as well as the professional competence needed to change the employment environment. Based on the boundaryless career theory (Sullivan, 1999), both working capabilities and professional competence would transform into employment competency to enhance employment competitiveness. The top three items are “learning skill,” “planning, coordination and organizational skill,” and “problem-solving skill,” with the total percentages of “much” and “a great deal” coming in at 73.2, 72.9, and 72.8%, respectively. In contrast, the improvement of “critical thinking skill” and “statistical and data-processing skill” are not obvious. The third cluster, “basic skills” (Figure 4), contains the level of knowledge personally possessed as well as the fundamental and generic skills for graduates to cope with sophisticated business. The top two items are “broad general knowledge” and “professional knowledge,” with the total percentages of “much” and “a great deal” being 76.0 and 74.5%, respectively. Conversely, the development for both “foreign language skill” and “financial literacy” is less achieved.

### Multivariable Linear Regression Analysis

We will now discuss the first hypothesis that educational practice has a significant positive impact on the development of finance and trade graduate employability. First, professional education emphasizes on strengthening graduates' professional qualification, which indicates from Tables 5–7 that professional education has a significant positive effect on the development of finance and trade graduate basic skills (0.433). The reasons for greater improvement in basic skills are because graduates lacked career recognition, job planning awareness, and professional orientation before entering college, and professional education accounted for their poor social and practical skills during college. Second, transferable education focuses on the cultivation of “integrative” thinking and “cross-fields” capability. Moreover, it indicates a significant positive effect on the enhancement of finance and trade graduates' basic skills (0.211). That means the development of graduate basic skills is closely connected to both professional education and transferable education. Third, teaching resources support places emphasis on providing graduates with all-round and three-dimensional teaching cultivation, practical guidance, and employment support. The model shows that it has a significant positive effect on the improvement of finance and trade graduates' soft skills (0.418), as well as professional skills (0.242). Therefore, professional education has a significant positive impact on finance and trade graduates' basic skills, transferable education has a significant positive impact on graduate basic skills, and teaching resources support has a significant positive impact on both soft skills and professional skills.

**Table 5:** Factors impact on finance and trade graduates' soft skills.

**Variables**	**Soft skills**	
	**B**	**VIF**
Gender: Male	0.127^*^	1.106
Family structure: more than one child	−0.019	1.334
Family yearly income per capita: RMB 3,001–5,000	0.028	1.675
Family yearly income per capita: RMB 5,001–10,000	0.102	1.767
Family yearly income per capita: RMB 10,001–20,000	−0.071	1.644
Family yearly income per capita: RMB 20,001–50,000	0.270^**^	1.452
Family yearly income per capita: Above RMB 50,001	0.180	1.685
Family location: provincial capital and municipalities	0.163	1.673
Family location: province administrative cities	−0.120	1.532
Family location: county towns	0.001	1.647
Family location: townships	−0.209^*^	1.355
Fathers' education years	−0.002	2.189
Mothers' education years	0.022^*^	2.114
Fathers' occupation: managerial and technical staff	−0.126	1.746
Mothers' occupation: managerial and technical staff	−0.130	1.666
Learning length	0.023^**^	1.125
Participate in minor courses/dual degrees	0.153	1.053
Qualification certificates: obtained (language, computer, and vocational skills)	0.027	1.072
Internship engagement	0.002^*^	1.143
Clubs activities engagement	0.001	1.149
Social networking length	0.010	1.054
Student-related matters: student leader	−0.044	1.136
Academic scores: middle 25–50% in major	0.063	2.133
Academic scores: top 25% in major	0.124	2.309
Scholarships: obtained	0.142^*^	1.095
Professional education	0.098	8.222
Transferable education	−0.071	5.554
Teaching resources support	0.418^***^	4.501
Constant	−0.653^***^	

*** *p < 0.01*.

** *p < 0.05*.

* *p < 0.1*.

**Table 6:** Factors impact on finance and trade graduates' professional skills.

**Variables**	**Professional skills**	
	**B**	**VIF**
Gender: male	0.112	1.106
Family structure: more than one child	−0.081	1.334
Family yearly income per capita: RMB 3,001–5,000	−0.057	1.675
Family yearly income per capita: RMB 5,001–10,000	0.047	1.767
Family yearly income per capita: RMB 10,001–20,000	0.064	1.644
Family yearly income per capita: RMB 20,001–50,000	0.094	1.452
Family yearly income per capita: above RMB 50,001	0.048	1.685
Family location: provincial capital and municipalities	0.033	1.673
Family location: province administrative cities	−0.001	1.532
Family location: county towns	0.030	1.647
Family location: townships	−0.017	1.355
Fathers' education years	−0.007	2.189
Mothers' education years	0.006	2.114
Fathers' occupation: managerial and technical staff	0.037	1.746
Mothers' occupation: managerial and technical staff	−0.030	1.666
Learning length	0.015	1.125
Participate in minor courses/dual degrees	−0.186^*^	1.053
Qualification certificates: obtained (language, computer, and vocational skills)	−0.075	1.072
Internship engagement	0.003^**^	1.143
Clubs activities engagement	0.006^**^	1.149
Social networking length	0.002	1.054
Student-related matters: student leader	0.003	1.136
Academic scores: middle 25–50% in major	−0.137	2.133
Academic scores: top 25% in major	0.007	2.309
Scholarships: obtained	0.038	1.095
Professional education	0.079	8.222
Transferable education	0.106	5.554
Teaching resources support	0.242^***^	4.501
Constant	−0.213	

*** *p < 0.01*.

** *p < 0.05*.

* *p < 0.1*.

**Table 7:** Factors impact on finance and trade graduates' basic skills.

**Variables**	**Basic skills**	
	**B**	**VIF**
Gender: male	0.043	1.106
Family structure: more than one child	0.011	1.334
Family yearly income per capita: RMB 3,001–5,000	−0.045	1.675
Family yearly income per capita: RMB 5,001–10,000	−0.087	1.767
Family yearly income per capita: RMB 10,001–20,000	0.027	1.644
Family yearly income per capita: RMB 20,001–50,000	−0.347^***^	1.452
Family yearly income per capita: above RMB 50,001	0.004	1.685
Family location: provincial capital and municipalities	−0.071	1.673
Family location: province administrative cities	0.044	1.532
Family location: county towns	0.013	1.647
Family location: townships	0.189	1.355
Fathers' education years	0.006	2.189
Mothers' education years	−0.013	2.114
Fathers' occupation: managerial and technical staff	−0.048	1.746
Mothers' occupation: managerial and technical staff	0.234^**^	1.666
Learning length	−0.036^***^	1.125
Participate in minor courses/dual degrees	0.101	1.053
Qualification certificates: obtained (language, computer, and vocational skills)	0.044	1.072
Internship engagement	0.001	1.143
Clubs activities engagement	0.001	1.149
Social networking length	−0.009	1.054
Student-related matters: student leader	0.000	1.136
Academic scores: middle 25–50% in major	0.086	2.133
Academic scores: top 25% in major	0.064	2.309
Scholarships: obtained	−0.099	1.095
Professional education	0.433^***^	8.222
Transferable education	0.211^***^	5.554
Teaching resources support	−0.093	4.501
Constant	0.126	

*** *p < 0.01*.

** *p < 0.05*.

^*^p < 0.1.

Regarding the second hypothesis that student engagement has a significant positive impact on the development of finance and trade graduate employability, as can be seen in Tables 5–7, learning length has a positive influence on finance and trade graduates' soft skills (0.023); however, it caused an impediment in basic skills development (−0.036). By devoting time and energy to study, finance and trade graduates have the chance to develop their independence and concentration, as well as their teamwork ability, leadership, and loyalty. However, longer learning length experience has a negative influence on basic skills, which indicates that the accuracy and efficiency of learning for higher vocational students need to be reevaluated and improved. Internship engagement has a positive influence on soft skills (0.002), and it is obvious that for higher vocational education, doing and practicing could enhance finance and trade graduates' professional skills and job competency. Meanwhile, the more internship engagement graduates get involved in, the better their professional skills (0.003). In addition, finance and trade graduates who pay more attention to club activities could also enhance their professional skills (0.006), which represents their strong coordination and innovation abilities. However, graduates participating in minor courses or obtaining dual degrees has a negative influence on the development of their professional skills (−0.186).

Regarding the third hypothesis that family background has a significant positive impact on the development of finance and trade graduate employability, Tables 5–7 show that only family income per capita, which is between RMB 20,001 and 50,000, has a positive influence on graduates' soft skills (0.270) development while it weakens their basic skills (−0.347); finance and trade graduates from townships present weaker soft skills (−0.209), indicating less professional education resources or opportunities provided to develop their capabilities. Meanwhile, mothers play a more important role for graduates; their education years have a positive influence on graduates' soft skills (0.022). Meanwhile, the mother's occupation as a managerial or technical staff member has a positive impact on their basic skills (0.234).

## Discussion

This study treats employment as an ongoing process, focusing on the development and sustainability of graduates. According to the survey, soft skills, professional skills, and basic skills have been presented to assess finance and trade graduate employability. In general, with the development of globalization, emerging industries such as *New Retail, Artificial Intelligence, Online Marketing*, and *Big Data*, have brought great challenges to finance and trade graduates. In order to blend into the competitive and sophisticated employment market, graduates need to possess strong competence to conduct business group tasks and obtain skilled accounting data-processing ability, logical and critical market analyzing skills, and strong financial literacy. However, currently, these knowledge and skills are just their weaknesses; hence, measures need to be assessed and taken to overcome these difficulties. As finance and information technology have become the top industries of choice for employment among college graduates (Yue and Zhou, 2019), the labor market could provide plenty of major-related opportunities for finance and trade graduates. Under these circumstances, the following three factors affecting employability can be summarized.

College educational practice plays the most important role in developing finance and trade graduate employability. Some educational reforms have aimed at market-orientated approaches to enhance graduate employability or emphasized the effectiveness of the higher vocational education system in responding to market needs. The findings imply that soft skills and basic skills have immensely improved through educational practice, such as providing professional projects, occupational knowledge and skills training, off-campus internships, and other practical programs to enhance their career competence. Work placement periods during study could develop graduate employability and cultivate graduates with specific skills particularly in connection with the workplace (Bonnard, 2020); extracurricular activities (Roulin and Bangerter, 2013) are also significant in graduates' future academic and professional careers. In contrast, transferable education has great potential to inspire finance and trade graduates to be more qualified and competitive among candidates. This is due to the rapid growth of the world economy, which has come up with many new technologies, products, or services around the world; accordingly, finance and trade graduates will face unprecedented challenges and risks to survive in this competitive business context. Principally, those who just focus on their major-related field are not qualified for the employment market, so, they ought to enhance the ability to transfer their knowledge and skills into brand-new or unfamiliar fields to demonstrate their strong competence. As graduate employability is not just a mere collection of skills, knowledge, and personal qualities, it could be considered as a form of self-transformation (Hall, 2019) in the labor market. Hence, multidisciplinary knowledge or integration of general skills becomes vital for graduates, and transferable education undertakes an irreplaceable responsibility, because finance and trade graduates' learning skills and problem-solving skills are essential for them to take on difficult tasks. College education is also like a bridge to link graduates and the labor market by providing more career guidance or authentic training and facilitating the implementation of educational support. As a result, attention must be placed on the outcomes of college educational practice.

Student engagement is an essential factor in promoting finance and trade graduate employability development. Graduates with high motivation for learning could enhance their soft skills, and more internship or club activities' engagement brings stronger professional skills. Graduates' interests in teaching content could motivate them to engage in learning (Evertson, 2015; Ertel, 2021); meanwhile, Pedler et al. (2020) constructed a tri-dimensional frame of student engagement, based on the findings of Fredricks et al. (2004), which emphasizes on the teacher's role in promoting positive student engagement, containing graduate actions/observable behaviors, feelings/internal emotions, and thoughts/internal cognitions. The learning length for core subjects or optional ones could indicate graduates' attitude to study as well as their strong will to gain more knowledge, which pushes them to devote themselves to a deeper study of academic theory and method. Meanwhile, graduates who participated in multi-course or multi-degree learning could reflect their proper learning method or management—for example, the time, energy, attention, or other resources to fulfill the courses. Thus, the combination of learning attitude, motivation, strategy, and management enriches the learning character, which is the basis for future career development. As for social engagement, higher vocational colleges pay more attention to realistic practice or training to enhance graduates' manipulative or operational ability and it is also necessary to assess the relevance of the targeted internship or activities. For finance and trade, practical abilities of accounting, cross-border e-commerce operation, or social media marketing are the professional skills that graduates need to possess. By accumulating experience, finance and trade graduates have the capability to get down to the real situation and solve the difficulties based on the lessons drawn from their practice, which demonstrates their competitiveness for the job.

Different family backgrounds bring different enhancements to graduate employability, and this factor has the least effect compared to educational practice and student engagement. Finance and trade graduates could have more opportunities or broader pathways to get in touch with versatile learning materials or resources in families with more than RMB 20,001-50,000 yearly income, which significantly boosts their soft skills development. These findings are similar to Peking University's large-scale questionnaire survey on graduate employment in 2003, which found that family background has a significant positive impact on graduate employment, especially the opportuinity to find a job and the starting salary (Wen, 2006); also, parents may increase employment opportunities through informal networks (Sylos Labini, 2008), acting as a “network provider.” Townships provide a less effective soft skills practice channel and fewer opportunities for graduates, perhaps because of the weaker regional industry-education integration or effective internship programs cooperating with local enterprises. Moreover, the more years of education the mother receives, the less likely that the graduates will choose grassroots employment (Wang and Wen, 2015). In this study, the growth and development of finance and trade graduates are more likely to be connected with their mothers. This means that the learning habits, attitude toward work, flexibility, or other personal qualities could be taught by the mother, while basic knowledge or skills could also be strengthened during daily interactions.

Therefore, the research in this article provides valuable insights into the employability analysis, supporting hypotheses H1, H2, and H3. The results of the verification of the research hypotheses are presented in Table 8.

**Table 8:** Research hypothesis.

	**Research hypothesis**	**Result**
H1	Educational practice has a significant positive impact on the development of finance and trade graduate employability.	Accept
H2	Student engagement has a significant positive impact on the development of finance and trade graduate employability.	Accept
H3	Family background has a significant positive impact on the development of finance and trade graduate employability.	Accept

## Conclusion

To conclude, teaching resources support has a significant positive effect on finance and trade graduates' soft skills and professional skills of higher vocational colleges, and professional education and transferable education have a significant positive effect on their basic skills. Learning length, internship engagement, club activities' engagement, family yearly income per capita of RMB 20,001–50,000, mothers' education years, and mothers' occupation as a managerial and technical staff have a significant positive influence on graduate employability.

Therefore, this study contributes to the conceptual evolution of employability, especially for finance and trade college graduates. It is recommended for higher vocational colleges to continuously seek sufficient and effective educational practices to enhance finance and trade graduate employability and create an active and flexible learning environment to promote their engagement. For example, college and industry collaborations—in this case, enterprises such as cross-border e-commerce companies, joint-venture banks, international forwarding companies, or social media platforms— could participate in the course planning, technical training, distance seminars, lectures, workshops, conferences, or comprehensive projects with colleges. Furthermore, collaboration could occur *via* multidisciplinary activities. Resources, including equipment, technology, methods, manpower, and facilities, can be shared among parties for mutual benefits in the long run. This can lead to a great enhancement of finance and trade graduate employability as well as higher vocational education quality.

### Implications for Theory

This study helps to compare with foreign quantitative research experience and enrich the relevant theories of the employability development model of finance and trade graduates in higher vocational colleges; moreover, the influencing factors affecting the development of finance and trade graduate employability have been identified and the internal mechanism of educational practice on the development of graduate employability has been analyzed for better understanding of the connotation construction of higher vocational education, which have important theoretical significance.

### Implications for Practice

First, it helps us to understand the connotation and influencing factors of the employability of finance and trade graduates in higher vocational colleges so as to point out the direction for the effective promotion of higher vocational teaching reform. Second, it can not only help graduates achieve their personal sustainable career development, but also contribute to the practice of social responsibility conducted by industries and enterprises. Third, higher vocational education is inseparable from the labor market, as employment-oriented research could result in providing reference for our future education reform polic-making.

## Limitations and Further Research Opportunities

The issues of graduate employability in higher vocational colleges are gaining significant focus in education literature. This study focuses on the current attention and attempts to obtain a better understanding of employability factors. The findings of this study are subject to several limitations. For example, the measurement of employability may not accurately highlight the updated competence for finance and trade graduates as the knowledge, skills, values, and qualifications are upgrading. Future research should consider in-depth employability for finance and trade graduates. Furthermore, we did not consider employers' perceptions of finance and business graduate employability; thus, it would be useful if further studies could discover the opinions of employers as well as the industrial requirements to enhance the analysis of the factors affecting college graduate employability.

## Data Availability Statement

The raw data supporting the conclusions of this article will be made available by the authors, without undue reservation.

## Ethics Statement

The studies involving human participants were reviewed and approved by Peking University. The patients/participants provided their written informed consent to participate in this study. Written informed consent was obtained from the individual(s) for the publication of any potentially identifiable images or data included in this article.

## Author Contributions

XH and GZ described and developed the review and the hypotheses. XH, JC, GH, and XC were involved in the data collection process. XH, ZL, JC, GH, and XC performed the analysis, interpretation of the results and formulated the main conclusions. ZL and GZ formulated the study limitations and future directions for the research. All the authors' help editing and formatting the paper.

## Funding

This work was supported by the key project of the National Social Science Fund of China – the Research on the Promotion Mechanism of College Students' Employment and Entrepreneurship Ability in the Digital Era (21ASH008) and Professional Development Program for visiting Scholars of Higher Education 2021 – the Research on the Promotion Mechanism of Employability of Higher Vocational College Graduates in Digital Era.

## Conflict of Interest

The authors declare that the research was conducted in the absence of any commercial or financial relationships that could be construed as a potential conflict of interest.

## Publisher's Note

All claims expressed in this article are solely those of the authors and do not necessarily represent those of their affiliated organizations, or those of the publisher, the editors and the reviewers. Any product that may be evaluated in this article, or claim that may be made by its manufacturer, is not guaranteed or endorsed by the publisher.
